# Connection of reactive oxygen species as an essential actor for the mechanism of phenomena; ischemic preconditioning and postconditioning: Come to age or ripening?

**DOI:** 10.14744/nci.2021.78466

**Published:** 2021-12-31

**Authors:** Demet Sengul, Ilker Sengul

**Affiliations:** 1. Department of Pathology, Giresun University Faculty of Medicine, Giresun, Turkey; 2. Division of Endocrine Surgery, Giresun University Faculty of Medicine, Giresun, Turkey; 3. Department of General Surgery, Giresun University Faculty of Medicine, Giresun, Turkey

**Keywords:** Ischemia-reperfusion, ischemic postconditioning, ischemic preconditioning, reactive oxygen species, reperfusion injury

## Abstract

Ischemic preconditioning (IPC), in 1986, and then ischemic postconditioning (IPoC), in 2003, were determined and lots of studies on the many organs were performed about the preventive effects of these strong endogenous mechanisms on the relevant tissues against ischemia-reperfusion and their protective impressions have been emphasized by many authorities up to date. Reactive oxygen molecules are immensely active molecules, originating from molecular oxygen, playing a principal role in intracellular signalization, aging, and various pathologic conditions. Reactive oxygen species (ROS) such as superoxide anion and hydrogen peroxide are known in the pathogenesis of ischemia-reperfusion (I/R) injury. In the pathogenesis of cellular and tissue injury in I/R, the significant output of ROS in the initial phase of reperfusion, particularly between the 1^st^ and 7^th^ min, has been propounded as being an essential and crucial main factor for the phenomena. Even though a great deal of mechanisms has been asserted for IPC and IPoC, the distinct shielder mechanism(s) was/were not clearly proved yet. However, occupying a significant place of ROS among these forecasted mechanisms has been advocated up to date.

**T**he action of previous short-term ischemia-reperfusion (I/R) periods, rendering the myocardium more resistant to latter main I/R injury was first described as “ischemic preconditioning (IPC)” by Murry et al. [1] in 1986. Afterward, the short-term repetitive brief episodes of I/R, carried out just after, instead of just before the exposed ischemia, were studied with a similar regimen first by the Vinten-Johansen group [2] in 2003 and this phenomenon, another strong endogenous mechanism, was termed as “ischemic postconditioning (IPoC).”

Although a variety of protective mechanisms have been held accountable for IPC, accessibility of potassium (K^+^) adenosine triphosphate (ATP) (KATP) channels in both the early and late phase of protection have been denoted [3, 4]. Some findings are extant about these channels serving as an end-affecting parameter. As intracellular ATP concentration decreases, KATP channels permit to egress by K^+^ opening that leads to attenuate calcium (Ca^2+^) entrance by curtailing the duration of an action potential. The mentioned event provides preservation of energy and mitigates the osmotic intumescence, secondary to ischemia [5].

## Clinical and Research Consequences

Even though all the protective mechanisms of IPoC have not been known precisely, the most being held accountable ones are delayed opening of the Ca^2+^-induced mitochondrial permeability transition pore (mPTP) in the internal mitochondrial membrane [6, 7]; activation of components of the reperfusion injury salvage kinase pathway, particularly phosphatidylinositol 3-kinase-Akt (also known as protein kinase B [PKB]) pathway, adenosine [8–10], nitric oxide-guanylate cyclase, protein kinase C, and PKB, and KATP channels [4, 8, 11, 12]; prevention of mitochondrial ATP-sensitive potassium (mKATP) channels mKATP channels, mitochondrial peroxide production, and glutathione (GSH), the main intracellular essential non-enzymatic agent [13]; inhibition of reactive oxygen (O_2_) species (ROS) production and intracellular excessive Ca^2+^ accumulation [14, 15]; and prevention of apoptotic, necrotic, and autophagic cardiomyocytic cell death [16]. Attenuation in the neutrophil accumulation, oxidative stress, apoptotic cell death, mitochondrial accumulation, osmotic gradient, and cell intumescence [17–19], sodium-hydrogen (Na/H) pump activation, myocardial contractions [20, 21] has been reported by way of these mentioned mechanisms in the early minutes of reperfusion [22]. Of these many targets, attenuation of ROS may be a major mechanism, because reduced oxidant generation would attenuate direct tissue injury as well as remove a major stimulus that opens the mPTP at reperfusion [6, 7]. ROS have been cognized as rather reactive molecules, possessing a significant role in the intracellular signalization and being derived from molecular O_2_ along with aging and various pathological processes [23].

### ROS and I/R Injury

The emphasis on superoxide anion (O·̅_2_), hydrogen peroxide (H_2_O_2_), nitric oxide (NO), and peroxynitrite (ONOO–), reactive O_2_, and nitrogen species, in the pathogenesis of I/R injury, has been well known [24]. The principal component of the cellular defense mechanism against free radical-induced tissue injury is known as reduced GSH, a tripeptide (γ-glutamyl-cysteinyl glycine) that contains a free thiol group, and it is utilized as an indicator of oxidative stress, produced by ROS during the I/R process. The predominant source of ROS is known as O·^®^_2_, appearing as an electron reduction product of O_2_. After its chemical reaction, H_2_O_2_ occurs, and then H_2_O_2_ is catalyzed to H_2_O and O_2_ or to highly reactive hydroxyl radical (OH) with Fenton reaction. Some certain centers of mitochondrial respiratory chain complex are O·^®^_2_ sources in quite a few tissues [25].

**Highlight key points**

• Of the many targets, attenuation of ROS might be a major mechanism in the early minutes of reperfusion of I/R injury by reducing oxidant generation that leads to alleviating direct tissue injury as well as removing a major stimulus that opens the mPTP at reperfusion.
• Excessive production of ROS in the reperfusion phase paves the way for tissue injury. As such, ROS levels are the outcome of the balance between the compensatory enzymes and the production rate.
• In spite of ROS having been recognized in the later periods, it has been suggested that the highest rates of production in the tissues occur in the 1^st^ and 7^th^ min from the onset of reperfusion.
• ROS has a crucial role in both the protective mechanisms and pathogeneses of IPoC and IPC.
• Many factors/substances have been charged in the pathogeneses and mechanism of the conditionings, but they have not been clarified yet.

The cell protective effect of K^+^ channel openers, described on the inner membrane of mitochondria, is being stimulated by the mitochondrial ROS production and the mitochondrial ROS production is being formed by these channel opener chemicals have been reported [25]. The first studied and get a high attention channel was mKATP channels [4]. Afterward, high K^+^ conductance (~300 pS), voltage-dependent, Ca^2+^-sensitive mitochondrial channel, and large-conductance Ca^2+^ -activated potassium (mitoBKCa) channels came into prominence [26]. However, the opinion of activation of K^+^ channels on the inner membrane of mitochondria, generally accepted, is about providing the transmission to the cellular matrix and its effect of attenuating the mitochondrial membrane potential and following respiratory rate, leading to the matrix alkalinization. Nevertheless, there is not any consensus about how mitochondrial K^+^ transmission affects the production of mitochondrial O·^®^_2_ [23].

I/R culminates with the genesis of ROS and that plays an important role in arriving at the tissues and attachment of the endothels for polymorphonuclear leukocytes that lead to tissue injury by means of increasing microvascular permeability. The cell injury induced by toxic O_2_ metabolites occurred by lipid peroxidation. It is reported that free radicals may trigger lipid peroxidation by reacting with cholesterol and unsaturated fatty acids [27].

### ROS and IPC

Lipid peroxidation and ileal epithelial apoptosis are accused of tissue injury in I/R. The results of IPC and I/R groups were evaluated and it was propounded that the ileal malondialdehyde (MDA) levels were increased significantly in I/R while this increment was precluded in IPC [27, 28]. Even though exceedingly raised ROS levels disrupt polyunsaturated membrane lipids by peroxidating their structure and functions by enhancing phospholipid membrane permeability and liquidity, their low levels may contribute potentially to the prevention of cellular structure and the relevant functions. Dezfulian and colleagues [29] propounded that the nitrite (NO^2-^) therapy, administrated after cardiac arrest, attenuates ROS generation, leading to a recovery in the cardiac and neurologic functions and prolongation of the survival through reversible inhibition of mitochondrial complex I. ROS levels are the outcome of the balance between the compensatory enzymes and the production rate. Zhou et al. [30] reported that the ROS, appearing during the short interval anoxia, inducing augmentation of antioxidant enzymes are responsible for the prevention of the myocytes against the anoxia and reoxygenation. Exposure to low concentrations of ROS may produce the useful impacts of IPC in the experimental cardiac I/R models. Therefore, ROS may contribute potentially to the IPC phenomenon.

Mitochondrion has a significant capability in augmentation of excess amount of Ca^2+^ in normoxic conditions. However, the issue of Ca^2+^ intake during ischemia has not been clarified yet. Some authors propelled the triggering for the opening of mPTP employing excess accumulation of Ca^2+^ and exceeding production of ROS, leading to starting crucial events in the early minutes of reperfusion while some asserted just a little amount of increase in the ischemic cardiomyocytes, sempre under the cytosolic level [6, 31, 32].

It is propounded that ROS triggers protection during the reperfusion phase rather than ischemia. Sedlic et al. [33] reported the partial attenuation of mitochondrial membrane potential was being reinforced the cardioprotective effect of anesthesia-induced IPC by inhibiting the excessive ROS production, leading to delay in the opening of mPTP and stimulating the cell survival. The mentioned decrease in the mitochondrial membrane potential was stated as the inhibition of, first, mitochondrial ROS production, then mitochondrial Ca^2+^ intake [33]. Dong et al. [34] reported a remote IPC in the extremities as preserving the spinal cord in a ROS-dependent way, not a neuronal one. The cardioprotective effect of NIM811 in IPC, a specific mPTP inhibitor, is well known. Simerabet et al. [35] observed a strong relationship between ROS and mKATP channels in the delayed cerebral IPC model and predicted the mitochondria as taking a central role in the neuroprotective effect.

Hepatic I/R injury occurs during the transplantation, trauma, and elective resection. It has been shown that ROS and apoptosis play significant roles in organ I/R injury. Calcitonin gene-related peptide (CGRP) is a noteworthy vasodilator, imitating the cardiopreserving action on the IPC model. CGRP is a basic sensory neurotransmitter, extensively located in the cardiovascular system. Song et al. [36] declared that CGRP therapy inhibited hepatic caspase-3 activity and apoptosis. In the mentioned study, the inhibitory effect of CGRP on the hepatic I/R injury occurred through a ROS-dependent pathway.

It is well known that the moderate O·^®^_2_ production, induced by K^+^ channel openers, leads to the beneficial effects of these openers on IPC. However, excessive production of ROS in the reperfusion phase paves the way for tissue injury. It was observed that this kind of injury decreased by the use of the treatment with K^+^ channel openers [37]. When the causative agent, leading to ischemia, is eliminated and the tissue is exposed to the reperfusion, reoxygenation is provided and this condition becomes a potent stimulant for the mitochondrial O·^®^_2_ entrance which leads to the cellular oxidative injury. Antioxidant therapy restricts these kinds of reperfusion-induced injury in the tissues and this verifies ROS playing a crucial role in these types of injuries [38].

### ROS and IPoC

As the main factor in the pathogenesis of tissue/cell damage, it is emphasized that the excessive amounts of ROS production lie in the initial period of reperfusion. In spite of ROS having been detected in the later periods, it has been suggested that the highest rates of production in the tissues occur in the 1^st^ and 7^th^ min from the onset of reperfusion [39, 40]. Argaud et al. [41] reported that the mitochondria isolated from the post-conditioned myocardium exhibited increased resistance to the Ca^2+^ loading. In other words, IPoC has been shown to “delay” the opening of mPTP, induced by Ca^2+^. Xia and Irwin [42] propounded that esmolol ceased the volatile anesthetic agent-induced IPoC by eliminating ROS. Penna et al. [43] declared that the intermittent activation of bradykinin B2 receptors and mKATP channels trigger the cardiac IPoC through a ROS signaling pathway.

It has been shown that intestinal IPoC prevents intestinal damage, albeit partial, by decreasing oxidative damage in the intestinal tissues. Liu et al. [44] emphasized the importance of early minutes of reperfusion for intestinal protection. However, Bretz et al. [45] reported a reverse view and argued that the IPoC was not effective in reducing I/R injury in the rabbit’s small intestines. Chu et al. [46] propounded that the reduction in the generation of ROS and the appearance of less oxidant-mediated injury were provided by intestinal IPoC. Rosero et al. [47] investigated the effect of IPoC on the intestinal mucosal expression of toll-like receptor-4 (TLR-4), included in the pathophysiology of organ damage after I/R. They emphasized the damage-associated molecular patterns, released by the injured tissue during the conditions of oxidative stress, along with the ROS were also capable of activating TLR-4 mediated responses. They reported that the small intestinal I/R injury was ameliorated using IPoC, exhibiting a more favorable inflammatory response, which may be attributed to the attenuated mucosal expression of TLR-4. Hence, recently, Chen et al. [48] indicated that the intestinal IPoC can ameliorate I/R injury by evoking autophagy, activating Akt pathway and nuclear factor erythroid 2-related factor 2, and inactivating glycogen synthase kinase-3 beta.

We studied the effects of IPoC on the intestinal I/R injury in an experimental rat model for five groups with three separate IPoC models in terms of the inhibition of the events in the early minutes of reperfusion. Herein, after 30 min of global ischemia applied to the superior mesenteric artery after laparotomy for (i) control (no intervention); (ii) IPoC-3 (three cycles of 10 s of reperfusion-reocclusion, 1 min total intervention); (iii) IPoC-6 (six cycles of 10 s of reperfusion-reocclusion, 2 min total intervention); and (iv) sham (laparotomy only) and left them for the 120 min of global reperfusion. We reported that the deterioration in the infarct area, serum total creatine kinase (CK); lipid peroxidation, tissue MDA; and ileum morphology, histopathological scoring; was attenuated with the use of IPoC models. Of note, the IPoC, especially IPoC-6, was effective in attenuating post-ischemic findings by decreasing the intestinal tissue MDA, serum total CK activity, the inflammation scores with total histopathologic injury scores of Chiu classification. Herewith, the IPoC models exerted a protective effect on the intestinal mucosa by reducing the mesenteric oxidant generation, lipid peroxidation, and neutrophil accumulation. Last but not least, the six-cycle algorithm demonstrated best protection for the intestinal I/R injury [49, 50].

### Conclusions

Of note, though IPC and IPoC have been studied in substantial and worthy fields in recent years, such as catheter-based reperfusion, cardiac surgery, and organ transplantation, the mechanisms of these phenomena have not been precisely established, yet. The researchers working with ROS, one of the most suspected mechanisms of the conditionings, are not fewer for IPoC than IPC.

To this end in many studies, the idea that ROS has a crucial role in both the protective mechanism and pathogenesis of IPoC and IPC has been supported to date. Last but not least, many factors/substances have been charged in the pathogenesis and mechanism of the conditionings but have not been clarified yet. This issue merits further investigation regarding their mechanisms and effectiveness to be able to illuminate the relevant cellular protection.
